# Notes and News

**Published:** 1904-04-09

**Authors:** 


### Notes and News.

The late Miss Elizabeth Wade, of Sheffield, has bequeathed £2,000 to the Sheffield Infirmary.

The first concert held in St. James's Hall, so soon to be demolished, was the opening concert, given in aid of the Middlesex Hospital.

It has been decided to extend the Coventry and Warwickshire Hospital, the work to be carried out by degrees as the finances of the institution permit.

The festival dinner of the Royal London Ophthalmic Hospital has been postponed to Tuesday, May 10, owing to the death of the Duke of Cambridge, who was patron of the hospital.

The late Mr. Edward Turner, of Newcastle-under-Lyme, has bequeathed £500 to the North Staffordshire Infirmary and £300 to the Longton Cottage Hospital. The late Miss Hannah Behrens has bequeathed £500 to the Cancer Hospital and £300 to University College Hospital.

The new Sanatorium for Infectious Diseases, at Scarborough, was opened recently. The site consists of seven acres, procured at a cost of £1,200. The Sanatorium consists of separate blocks for administration, scarlet fever, enteric fever, dipththeria, and observation; also for the laundry and the porter and discharge purposes. A special feature is the provision of private wards for paying patients. The cost amounted to £15,000.

The announcement that the Agent-General for Victoria is prepared to receive applications from qualified practitioners for the office of Inspector-General of the Insane for that State is a matter of considerable interest. Firstly the post is worth £1,500, but as the announcement states that the emolument is not to exceed that sum, it looks rather as if the post were to be thrown open to public tender. In other words, it might be assumed that Victoria wants the very best man it can get within or without, but that, with much wisdom, it will only pay the highest fee if it attracts the right man.

A new infirmary has been opened in connection with the Holsworthy Workhouse. Provision is made for 15 patients, at a cost of £1,238.

A new wing has been opened at the York County Hospital, comprising nurses' quarters, operating theatre, outside fireproof staircases, and lift, involving an expenditure of between £7,000 and £8,000.

The operation theatre at the Liverpool Royal Infirmary, which has been entirely reconstructed through the generosity of an anonymous donor, is now open. The infirmary is now equipped with a theatre for each of its surgeons.

The Glasgow Western Infirmary is to have a new wing, containing accommodation for 59 patients, for resident medical men, and various offices. The extension scheme provides considerably more than this, but the immediate provision will entail an expenditure of upwards of £20,000.

Leicester is again under unpleasant observation, and those for and against vaccination will be claiming examples to establish their contentions from amongst the unfortunate victims of the small-pox, which has already obtained a serious hold in the town. Upwards of 20 cases have already been moved into the hospital.

The plague record in Johannesburg continues to swell, though the number of cases daily is small, and the latter ones have been entirely amongst natives. There is a good deal of plague scattered throughout the East, and Malta has declared as infected, Arabia (excepting Aden and Perim), China, India, Alexandria, and all ports on the Persian Gulf. At Lima, in Peru, 40 cases are recorded.

A new sanatorium has been opened at Leicester, after the pattern of American sanatoria for the purpose of restoring health, mainly through hydropathy. Baths of all kinds are a strong feature in the establishment, where provision is made also for various methods of physical culture through the medium of massage, Swedish treatment, and dieting. The residue, after paying expenses, is to be devoted to the treatment of poor patients.

The first section of the new buildings of the Royal Waterloo Hospital for Children and Women, Waterloo Bridge Road, S.E., will be available for the reception of patients by the second week in April. A jumble sale will be held in the new out-patients' department at 3.30 p.m. on Saturday, April 9th. Any contribution of cast-off clothing, crockery, carpets, or furniture, will be most gratefully received.

A new Isolation Hospital for the Hampton District Council has been opened in the Uxbridge Road. The accommodation is for 10 patients. The blocks are over 40 feet apart, and there is plenty of room for extension on the site. The air space per bed is 2,028 cubic feet. The offices are detached from the wards, but accessible by a covered way. An ambulance and mortuary are provided. The cost of the hospital has amounted to £3,400.

